# Fronto-striatal circuits in response-inhibition: Relevance to addiction

**DOI:** 10.1016/j.brainres.2014.09.012

**Published:** 2015-12-02

**Authors:** Sharon Morein-Zamir, Trevor W. Robbins

**Affiliations:** a Behavioural and Clinical Neuroscience Institute, University of Cambridge, Cambridge CB2 3EB, UK; b Department of Psychology, University of Cambridge, Cambridge CB2 0SZ, UK

**Keywords:** Stimulant dependence, Addiction, Stop-signal, Cognitive control, Drug use, Relapse

## Abstract

Disruptions to inhibitory control are believed to contribute to multiple aspects of drug abuse, from preexisting vulnerability in at-risk individuals, through escalation to dependence, to promotion of relapse in chronic users. Paradigms investigating the suppression of actions have been investigated in animal and human research on drug addiction. Rodent research has focused largely on impulsive behaviors, often gauged by premature responding, as a viable model highlighting the relevant role of dopamine and other neurotransmitters primarily in the striatum. Human research on action inhibition in stimulant dependence has highlighted impaired performance and largely prefrontal cortical abnormalities as part of a broader pattern of cognitive abnormalities. Animal and human research implicate inhibitory difficulties mediated by fronto-striatal circuitry both preceding and as a result of excessive stimulus use. In this regard, response-inhibition has proven a useful cognitive function to gauge the integrity of fronto-striatal systems and their role in contributing to impulsive and compulsive features of drug dependence.

*This article is part of a Special Issue entitled SI:Addiction circuits*.

## Highlights

• Human and animal response-inhibition can inform substance dependence theorizing.
• Response-inhibition difficulties contribute to impulsivity and compulsivity.
• Response-inhibition can serve as a useful measure of fronto-striatal integrity.

## 1 Introduction

The ability to suppress inappropriate behaviors is a hallmark of executive functions, and essential for adaptive control of everyday behavior. The psychological construct of response-inhibition has been applied broadly in several contexts but particularly to the psychological functions pertaining to overriding a planned action, or stopping a repetitive maladaptive behavior (Bari and Robbins, 2013). Investigations of response-inhibition have, to date, yielded important insights for understanding addiction disorders. Such insights into the psychological and neural processes mediating response-inhibition not only provide more refined models of drug dependence and behavioral addiction but also inform mechanism driven targets for future therapies. We focus largely on stimulant drug dependence as much of the impetus for research in this area has arisen from the study of animal models with translational relevance. We will initially provide a non-exhaustive survey of this field before focusing on translation to human drug abusers.

## 2 Response-inhibition: Translation from animal models

The psychological construct of response-inhibition has been influential in at least two aspects of the animal literature pertaining to the neurobiology of drug addiction. Both impulsivity and compulsivity require the notion of response-inhibition. Impulsivity can be defined as risky or premature behavior, for example when it is necessary to wait for the appropriate signal to perform a prepared response, whereas compulsivity can be defined as maladaptive preservative behavior (Dalley et al., 2011). Drug abuse and dependence is associated in humans with an impulsive, risk-taking tendency, and one form of impulsivity has also been associated with the tendency to exhibit binge intake of cocaine in rats (Dalley et al., 2007). Specifically, rats exhibiting high levels of premature responding in the 5-choice serial reaction time test of sustained attention also exhibit (i) reduced D2/3 receptor binding in the ventral striatum (Dalley et al., 2007), and (ii) altered grey matter in the nucleus accumbens core region (Caprioli et al., 2014). The fact that the impulsive behavior occurs *prior* to cocaine exposure suggests the possibility that at least some impulsivity is not simply caused by drug abuse but can also be antecedent to it (i.e. impulsivity is an ‘endophenotype’, see below).

The most parsimonious explanation of this deficit is that behavioral inhibition impairment is caused by a malfunctioning nucleus accumbens. However, other data indicate that the role of the nucleus accumbens in modulating this premature responding is influenced by both ‘top-down’ and ‘bottom-up’ neural influences: structures contributing to ‘top-down’ control include the infralimbic cortex and cingulate cortex (Chudasama et al., 2003) whereas the ascending 5-HT and noradrenergic pathways contribute to ‘bottom-up’, modulatory regulation (Economidou et al., 2012). Evidence for the noradrenergic influence is provided by the ameliorative effects of the selective noradrenaline reuptake inhibitor atomoxetine, whether either administered systemically or when infused into the nucleus accumbens shell region (Economidou et al., 2012).

The high impulsive rats also show impairments of impulsive choice; that is, they consistently choose small immediate food rewards rather than larger, delayed ones (Robinson et al., 2009), similarly to rats with excitotoxic lesions of the nucleus accumbens core (Cardinal et al., 2001). However, the possibility of a generalized response inhibitory deficit in these high impulsive rats is negated by the fact that they do not have slower stop-signal reaction times in the stop-signal task (SST) measuring a different type of impulsivity (Robinson et al., 2009), which may be mediated by dorsal striatal rather than ventral striatal mechanisms (Eagle et al., 2008, Eagle and Baunez, 2010).

This work on impulsivity is also relevant to research on relapse, where exposure to drugs, stress or conditioned stimuli may elicit drug-taking and drug-seeking behavior (Stewart, 1984). There are obvious parallels to the work on impulsivity as shown by evidence that (i) atomoxetine also reduces the reinstatement of cocaine—taking in rats following punishment-induced abstinence (Economidou et al., 2012) and (ii) a rich literature indicates a prominent role of the rodent infralimbic cortex in the reinstatement of drug-seeking behavior after its extinction (Kalivas and McFarland, 2003, Peters et al., 2008). Presumably both impulsivity and reinstatement result in part from behavioral disinhibition.

## 3 Reinstatement of drug-seeking behavior in rodents

The classic phenomenon is the reinstatement of drug-seeking behavior following its extinction by stress, drug primes or conditioned stimuli (Shaham et al., 2003). The paradigm has been criticized for its lack of translational validity, given that extinction of human drug-taking does not usually occur. Rather, drug-taking is customarily suppressed by punishment (e.g. prison) or voluntary abstinence. However, each of these situations implicates response inhibitory processes—extinction, punishment and volitional control, and it is intriguing to consider whether they are mediated by common, or at least, overlapping circuitry, such as can be revealed by functional imaging studies in humans (see below). An obvious problem in attempting this translation is the exact homological parallels that may exist for human and rodent prefrontal cortical circuitry. Whereas, for example the ventrolateral prefrontal cortex (PFC) is commonly implicated in self-control in humans, it is notoriously difficult to find the rodent homologue of this structure; whether it would reside in the rodent medial PFC ‘column’ or alternatively, in the more lateral orbitofrontal cortex, is difficult to ascertain.

For drug-primed relapse in rodents, manipulations of the ventromedial PFC, such as inactivation by a mixture of GABA-A and GABA-B agonists (muscimol and baclofen), identify the prelimbic cortex to be the major controlling influence over the nucleus accumbens core, which, again, appears to be a key structure that drives the disinhibited drug-seeking behavior (Kalivas and McFarland, 2003). Moreover, infusions of a 5-HT2C agonist into the rodent infralimbic cortex inhibit reinstatement, somewhat paralleling evidence that infra-medial PFC infusions of a 5HT-2A receptor antagonist block high levels of impulsive responding on the 5-choice task (Winstanley et al., 2004), above.

5-HT mechanisms are involved in the regulation of glutamate release and the latter has been a focus of possible therapeutic intervention to prevent relapse, for example with *N*-acetylcysteine, a cysteine pro-drug that indirectly increases extracellular glutamate (Zhou and Kalivas, 2008, Amen et al., 2011) and modafinil, an atypical stimulant drug that also reduces the reductions of glutamate that normally occur during relapse (Mahler et al., 2014). Recent work increasingly implicates glutamate mechanisms within the nucleus accumbens as a possible target for therapeutic agents such as mGluR agonists. Other elicitors of relapse such as conditioned cues, perhaps unsurprisingly, involve additional circuitry, for example, related to discrete conditioned and contextual cues (basolateral amygdala and hippocampus) and stress (bed nucleus of the stria terminalis), although it is likely that prefrontal inhibitory control mechanisms remain important also. However, there may be differences among drug classes (including opiates and alcohol, as well as stimulants see Bossert et al., 2013) and so it would be unwise at this point to claim that a restricted set of medial PFC projections mediate global forms of inhibitory response control.

An additional source of complexity is provided by models of compulsive drug-seeking, which can be gauged most readily when self-administration behavior proceeds despite obvious adverse consequences, such as punishment by electric foot-shock (Belin et al., 2008, Pelloux et al., 2012). Presumably such behavior also entails a change in response-inhibition processes probably mediated by PFC structures. Given that compulsive behavior implicates orbitofrontal (OFC)-striatal rather than medial PFC mechanisms (see Dalley et al., 2011), it would appear that the control of relapse and compulsive drug-seeking may be governed by different neural systems, although probably organized along the same general lines of top-down prefrontal control over striatal outflow.

## 4 Response-inhibition in human research

**Fig. 1:**
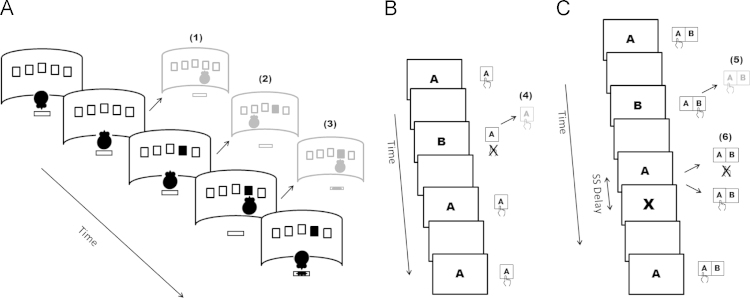
Panel 1a. Schematic representation of a single trial in the 5-CSRT task. The rat begins the trial with a nose poke in the food magazine. Following an intertrial interval (ITI), a brief light appears in one of the apertures and the rat must make a nose poke response in the appropriate hole in order to subsequently collect its reward. Premature responding occurs when the rat responds with a nose poke during the ITI rather than waiting (1). Error responding occurs when the rat responds to the wrong hole (2) and preservative responding occurs when it continues to respond rather than collect its reward. Panel 1b. Schematic representation of a sequence of trials in the go/no-go task. Subjects respond to one set of stimuli (‘A’) while withholding responses to another set (‘B’). Commission errors occur when subjects respond to no-go stimuli (4). Panel 1c. Schematic representation of a sequence of trials in the stop signal task. Subjects respond to go stimuli (‘A’ and ‘B’) presented on each trial. On a minority of trials a stop signal (in this case a visual ‘X’) indicates the prepotent response is to be withheld. As stop signal (SS) delay is varied so is the resulting probability of successfully inhibiting a response (6). Errors occur when subjects respond to a go stimulus by selecting the wrong key (5). By using a race horse model in combination with mean reaction time on go trials as well as the proportion of successful inhibitions and SS delay, an estimate of the latency to inhibit responding can be calculated (stop signal reaction time).

Response-inhibition in human research is relevant to many tasks requiring executive control, which in turn encompasses a more abstract inhibitory psychological construct in addition to cognitive flexibility and updating of working memory (Miyake et al., 2000). In this regard, response-inhibition has at times been considered to contribute to reversal learning and Stroop performance, which involve additional complex processes related to reward sensitivity, cognitive flexibility, rule learning and conflict resolution (Jentsch et al., 2014). Response-inhibition specifically has proven useful as measured by a relatively standardized set of paradigms that translate from experimental models and enable convergence across levels of analysis. These range from non-invasive and invasive imaging techniques in patients to lesion and pharmacological manipulations (Bari and Robbins, 2013, Chambers et al., 2009). Response-inhibition has most commonly been investigated using go/no-go (GNG) and stop-signal tasks (SST) which, though not identical, overlap considerably (Eagle and Baunez, 2010). In both tasks, going becomes prepotent by having prevalent speeded responses and infrequent no-go or stop stimuli. In GNG tasks, subjects respond to go stimuli whilst withholding responses to another set of no-go stimuli, yielding the key measure of commission errors, somewhat akin to premature responding. In the SST, subjects are encouraged to respond to go stimuli on all trials. However, these go stimuli are sometimes immediately followed by a stop stimulus indicating the planned response should be countermanded. The key measure of stop signal reaction time is derived by implementing a race-model (Logan, 1994). Other primary measures include execution latency and accuracy. Indices of error monitoring and strategic adjustments can also readily be derived Fig. 1.

**Fig. 2:**
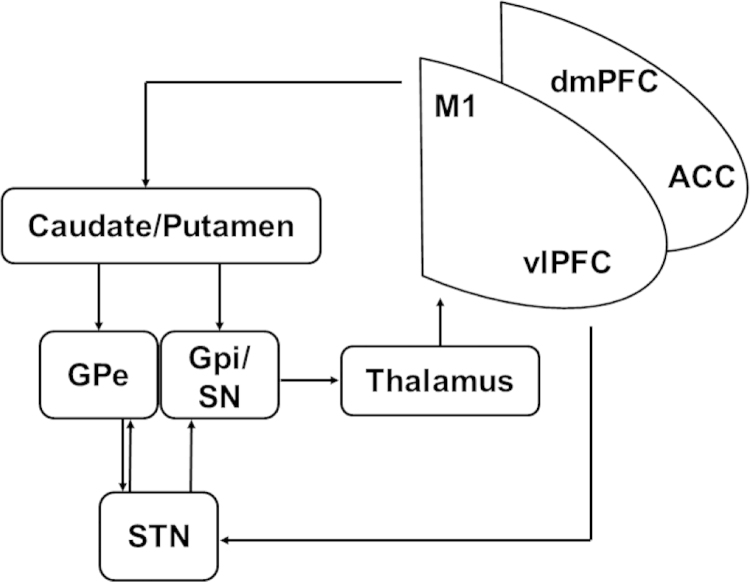
Schematic representation of circuitry involved in response inhibition including interactions between cortical areas as well as interactions with basal ganglia structures projecting via the thalamus back to the prefrontal cortex. M1 primary motor cortex; dmPFC dorsomedial prefrontal cortex (including the supplementary motor area); vlPFC ventrolateral prefrontal cortex (including the anterior insula and inferior frontal gyrus); ACC anterior cingulate, Globus pallidus pars externa GPe; Globus pallidus pars interna/reticular substantia nigra GPi/SN; subthalamic nucleus STN.

Considerable evidence implicates fronto-striatal circuits in response-inhibition, particularly as measured by GNG and SST (Bari and Robbins, 2013, Chambers et al., 2009). Prefrontal cortical involvement is robustly supported by human research, involving lesion, imaging, transcranial magnetic stimulation and electrocorticography data (Aron et al., 2003, Swann et al., 2012, Swick et al., 2011). The findings point to a circuit including the pre-supplementary motor area (SMA) in the dorsomedial PFC (dmPFC), and the anterior insula and inferior frontal gyrus in the ventrolateral PFC (vlPFC), particularly on the right (Floden and Stuss, 2006, Levy and Wagner, 2011). Subcortical involvement in response-inhibition involves direct and indirect loops in the striatum to the globus pallidus in addition to a parallel pathway via the subthalamic nucleus (STN) outputting to thalamocortical projections (Chambers et al., 2009, Eagle et al., 2008) (Fig. 2). Pharmacological manipulations in humans as in rodents have highlighted dopamine and noradrenaline involvement (Bari and Robbins, 2013, Eagle et al., 2008). Fronto-striatal loops involving the OFC and vmPFC do not appear to be primarily involved in suppressing inappropriate actions in such paradigms (Robbins et al., 2012), although their links to response-inhibition can be gauged in large scale studies (Whelan et al., 2012). In the remainder of the review, unless otherwise specified, we will use the term ‘fronto-striatal regions’ to describe those areas specifically involved in response-inhibition.

Over time, a refined model of response-inhibition has evolved acknowledging the relevance of multiple cognitive processes in GNG and SST. Thus, attentional monitoring and salience processing contribute to inhibitory performance with the inferior frontal junction together with parietal areas likely playing a role in detecting infrequent but behaviorally relevant stimuli (Luijten et al., 2014, Swick et al., 2011). Additionally, regions such as the dorsolateral PFC (DLPFC) are involved in rule and goal maintenance (Dosenbach et al., 2007). These tasks nevertheless engage a relatively narrow, overlapping and well-defined set of cognitive processes, providing a useful platform to explore more subtle control functions (Harle et al., 2014).

## 5 Response-inhibition and stimulant dependence

The rationale for investigating response-inhibition in stimulant dependent individuals (SDI) is clearly manifold. First and foremost, the symptoms manifested feature many aspects of impaired self-control and difficulties in inhibiting inappropriate behaviors. It is this loss of control that leads to persistence in drug-taking with larger amounts consumed for longer than intended, despite a desire to quit and despite adverse personal and social consequences (American Psychiatric Association, 2000). As noted above, motor disinhibition contributes to key aspects of impulsivity, as a core multifaceted construct promoting addiction (Dalley et al., 2011). Response-inhibition contributes to compulsivity, which is potentially orthogonal but also relevant to substance abuse (Lubman et al., 2004). In humans compulsivity is characterized by repetitive and ritualistic behaviors underscored by habit learning (Fineberg et al., 2014, Robbins et al., 2012). How increased compulsivity may contribute to the development of abuse is not presently well understood, but compulsivity becomes more prominent with chronic abuse as top-down control is continuously weakened and dorsal striatum-mediated habitual control becomes dominant (Everitt and Robbins, 2005, Everitt and Robbins, 2013). Beyond its specific and direct involvement in addiction, the neurocognitive substrates mediating response-inhibition also underlie aspects self, emotion and social regulation (Tabibnia et al., 2011, Volkow et al., 2011). Moreover, catecholamine regulation in these circuits is compromised in addiction (Goldstein and Volkow, 2011).

In accordance with such reasoning, many contemporary theories emphasize disrupted inhibitory control in drug use in combination with impulsive decision making and altered motivational and reward processes (Dalley et al., 2011, Goldstein and Volkow, 2011, Li and Sinha, 2008, Verdejo-Garcia et al., 2008). Disruptions to control processes contribute to multiple components of addiction such as increasing susceptibility to initial use, transition to dependence, maintenance, as well as contributing to relapse and difficulties in maintaining abstinence (Perry and Carroll, 2008).

There is broad agreement that response-inhibition is impaired in SDI (e.g., Li and Sinha, 2008, Luijten et al., 2014, Perry and Carroll, 2008, Spronk et al., 2013). Below we outline some main findings before considering emerging trends and issues. When discussing aberrant task performance and potential mediating neural substrates in SDI, key questions pertain to their causal role, their presence as a result of neurotoxic effects, or as an interaction between the two (Perry and Carroll, 2008). One approach is to examine available evidence in a framework outlining the temporal evolution of drug use, from prior to initial use to following chronic abuse, and in those who try to abstain: withdrawal, abstinence and at times, relapse.

Only a handful of papers have investigated response-inhibition in children or adolescents at risk for abuse for any substance. This is despite evidence from other paradigms of inhibitory control suggesting response-inhibition as a likely indicator of neurobiological risk for substance use disorders (Ivanov et al., 2008). In adolescents at risk for alcohol use disorders, stopping performance predicted not only alcohol-related problems but also illicit drug use independently of familial risk and attention deficit hyperactivity disorder (ADHD) and conduct disorder problems (Nigg et al., 2006). This evidence dovetails with another study where neurobehavioral disinhibition did not include response-inhibition measurements (Tarter et al., 2003, Tarter et al., 2004). Another approach has been to survey a large sample of adolescents, capturing the range of possible precursor behaviors or ‘behavioral endophenotypes’. Evidence from the IMAGEN project indicated that although adolescents who tried illicit substances did not differ in stopping performance from those who did not, they did exhibit higher activation in a right frontal network comprising vlPFC and ACC, thereby requiring greater brain activity levels to produce similar inhibitory performance (Whelan et al., 2012). Without longitudinal follow-up, it is unclear what portion of at risk groups went on to develop substance use disorders (but see Whelan et al., 2014, for initial longitudinal data).

A more substantial body of evidence speaks to worse response-inhibition performance in SDI with a mixed pattern for response execution. (Ersche et al., 2011, Ersche et al., 2012a, Fillmore and Rush, 2002, Hester and Garavan, 2004, Lane et al., 2007, Morein-Zamir et al., 2013, Morie et al., 2014a). Functional magnetic resonance imaging (fMRI) studies have generally reported reduced PFC activation accompanying response-inhibition difficulties, with hypoactivation of vlPFC and at times medial, cingulate, and DLPFC in addition to cerebrellar hyeractivation (Hester and Garavan, 2004, Kaufman et al., 2003, Morein-Zamir et al., 2013). The findings appear consistent despite differences across studies in the contrast used to isolate response-inhibition, and key procedural differences such as working memory load. Together, such converging-operations support the reliability of the accompanying hypoactivation. When considering brain structure, white matter integrity in key regions such as the vlPFC correlated with the response-inhibition performance in SDI, whilst no such robust relationships with grey matter volume were apparent (Ersche et al., 2012a). Although impairment is found consistently, its association with disorder severity measures is small and inconsistent. This is in keeping with other findings showing that executive dysfunction, as measured by laboratory tasks, is not necessarily associated in a straightforward manner with increased drug use (Morie et al., 2014a, Verdejo-Garcia and Perez-Garcia, 2007).

The relatively few reported abnormalities in the striatum may seem surprising given its role in response-inhibition and in mediating addiction in animal research. However, this is likely due to fMRI studies largely utilizing tasks that do not efficiently model response execution processes separately from baseline. Future studies may consider the benefits of introducing sequences that allow more sensitive assessment of striatal involvement. Additionally, evidence for anatomical striatal abnormalities in SDI has been particularly inconclusive, possibly due to its susceptibility to individual differences in recent history of abuse and/or abstinence (Ersche et al., 2013c).

Acute administration of cocaine or intravenous methylphenidate has led to improved response-inhibition performance in active or recently abstinent SDI (Fillmore et al., 2002, Fillmore et al., 2006, Garavan et al., 2008, Li et al., 2010), and occasionally also in response execution performance (Garavan et al., 2008). Concurrent fMRI indices indicate increased activation in the striatum, thalamus and PFC regions that are typically hypoactive in active users (Garavan et al., 2008, Li et al., 2010). This is consistent with a consensus that acute cocaine ameliorates or masks existing executive impairments (e.g., Woicik et al., 2009).

Given that inhibitory control probably plays a role in abstinence and relapse avoidance, it is significant that abstinent SDI show less robust patterns of impairment, suggestive of possible recovery or compensation. After short term to intermediate cessation of use (up to several weeks), response-inhibition appears impaired in some studies (Li et al., 2006b, Monterosso et al., 2005) but not others (Li et al., 2008, van der Plas et al., 2009). Response execution was not reported as significantly worse demonstrating a degree of specificity. Similarly, accompanying PFC hypoactivation appears less robust with reduced anterior cingulate (ACC) activation reported (Li et al., 2006b). Reduced vlPFC grey matter volume, worse inhibitory performance but no response execution differences was reported in abstinent methamphetamine users (Tabibnia et al., 2011). Evidence regarding performance in longer term abstinent users has been even more scarce in part due to the challenges of conducting such studies, which typically encompass a range of abstinence durations. Disruptions in response-inhibition were noted in some studies (Fernandez-Serrano et al., 2012) but not others (Bell et al., 2014a, Bell et al., 2014b, Verdejo-Garcia et al., 2006), with again a mixed pattern for response execution. Though one small study reported hyperactivation of some PFC regions (Connolly et al., 2012) this was not replicated subsequently (Bell et al., 2014a, Bell et al., 2014b). Electrophysiological aberrations during response-inhibition have also been reported (Franken et al., 2007, Morie et al., 2014b). However, the relationship between inhibitory control and such measures is complex and understanding their significance is still evolving (Huster et al., 2013).

In summary, the evidence points to response-inhibition deficits accompanied by reduced PFC recruitment in current SDI that ameliorates with acute stimulant dosing, and may normalize to some degree with abstinence. This conclusion is highly consistent with evidence from other forms of cognitive control in SDI involving fronto-striatal systems (Feil et al., 2010, Jovanovski et al., 2005). There remains a dearth of information regarding long-term abstinence and the role response-inhibition per se might play in its promotion, though if a preexisting vulnerability factor, it could be relatively unaffected by present state. This notion is consistent with evidence from other substance use disorders where response-inhibition appears unrelated to abstinence and treatment retention (Stevens et al., 2014). Alternatively, successful abstinence may involve active strengthening of inhibitory control, so that impairment in long-term successful abstainers may be paradoxically reduced compared to high-risk individuals. It remains unclear *how* precisely response disinhibition may serve as a risk factor for relapse though the mediating neural structures have been reported to be modulated by abstinence (Garavan et al., 2013).

Based on evidence from SDI cross-sectional studies, the degree to which neurobiological fronto-striatal abnormalities *predate or follow* stimulant abuse is unclear, though findings from at risk individuals indicate both are in play. Given the temporal evolution of addiction, it is only the difficulty of conducting longitudinal and prospective studies that has prevented their more pervasive use to help disentangle causal associations between cognition and drug use. Nonetheless, several such projects are set to shed light on how response-inhibition as part of a larger set of top-down control functions may typify stimulant use over time. Finally, it is possible that response-inhibition deficits, which progressively grow with age (Williams et al., 1999), may contribute even further to general impairment with increased cumulative use, consistent with evidence suggesting the deleterious effects of aging are accelerated in drug users (Ersche et al., 2013a).

## 6 Addressing the relationship between disinhibition and stimulant dependence

Relevant to the issue of the extent to which response-inhibition deficits predispose or result from stimulant dependence, are two additional lines of evidence. First, is the investigation of not only SDI but also their biological first degree siblings to detect common vulnerability markers. The notion that response-inhibition might prove a useful endophenotype, mediating genes and clinical symptoms (Gottesman and Gould, 2003) is plausible given that executive functions and brain structure are highly heritable (Friedman et al., 2008, Thompson et al., 2001), as is stimulant dependence (Merikangas and McClair, 2012). Closer inspection of characteristics unique to the unaffected siblings compared to the SDI and controls may also highlight protective or compensatory mechanisms (Morein-Zamir et al., 2013). Thus, response-inhibition performance in unaffected siblings has been reported as impaired compared to controls, acting as a shared trait in the sibling pairs (Ersche et al., 2012a). Moreover, response-inhibition performance was associated with white matter vlPFC abnormalities commonly shared in the sibling pairs (Ersche et al., 2012a). This shared vulnerability did not extend however to reduced prefrontal activation in the siblings, who demonstrated increased rather than decreased recruitment of key PFC regions including the pre-SMA (Morein-Zamir et al., 2013). The overall complex shared and distinct pattern of brain structure, personality traits and cognitive abnormalities support response-inhibition difficulties as being one of the most robust cognitive vulnerabilities predisposing to the development of stimulant drug dependence.

A second line of evidence is the investigation of recreational cocaine users, loosely identified by self-reported regular cocaine use, without fulfilling DSM-IV criteria. Response-inhibition disruptions were reported in recreational users in one small study (Colzato et al., 2007) though not in others (Harle et al., 2014, Vonmoos et al., 2013b), raising the question of whether adverse disinhibitory effects of regular stimulant use are inevitable. In another study, intact performance was accompanied by increased dmPFC and ACC activation suggesting inefficient neural recruitment (Morein-Zamir et al., submitted). Broader neurocognitive findings and self-reported impulsivity demonstrate similar patterns, with some studies indicating only subtle effects in recreational users (Ersche et al., 2013b) whilst others indicate them to be intermediate between controls and SDIs (Hulka et al., 2013, Vonmoos et al., 2013a, Vonmoos et al., 2013b). Recreational users, as SDI, consistently exhibit elevated sensation seeking (Mackey et al., 2014), a trait absent in unaffected siblings of SDI (Ersche et al., 2010), suggesting its orthogonality to response-inhibition. As recreational users typically present with fewer years and less cumulative use than SDIS, any differences might merely be due to presentation along different stages of the dependence trajectory (Preller et al., 2013). Alternatively, some recreational users may comprise a separate neurobiological phenotype, with normative cognitive, impulsivity and compulsivity levels along with increased rather than reduced OFC grey matter characteristic of SDI (Ersche et al., 2013b). Together with reports of controlled long-term usage and high intelligence, this suggests a resilient subgroup, in which any deleterious effects of repeated drug exposure are subtle. Increased characterization of potentially resilient individuals could inform not only therapeutic strategies but also enable more accurate animal models allowing better linkage to genetic and molecular mechanisms (Ersche et al., 2013b). However, with no consistent characterization of recreational or occasional users across studies, integrating findings remains a challenge.

Taken together, the findings elucidate how disruptions in response-inhibition contribute via multiple pathways to drug dependence. High impulsivity, manifested in part by impaired inhibition, hypothetically facilitates initial exposure and subsequent escalation, whilst elevated sensation seeking contributes only to the former. Elevated compulsivity likely contributes in parallel. Evidence that high impulsivity and to some extent compulsivity may predate drug use, is evidenced in the unaffected biological siblings (Ersche et al., 2012b). Age at onset of exposure comprises another critical factor, as response-inhibition is undergoing development when initial experimentation with drugs often occurs in SDI, whilst recreational users often experiment at a later age. Impaired response-inhibition in SDI may also contribute to maladaptive patterns of use, such as bingeing. In contrast, preserved response-inhibition in recreational users allows more controlled and sustainable usage, providing a degree of protection from the adverse consequences of protracted drug use. Inefficient recruitment of key fronto-striatal regions mediating response-inhibition is evident in the unaffected siblings, the recreational users and in adolescents reporting what could be construed as preliminary exposure (Morein-Zamir et al., 2013, Whelan et al., 2012). This suggests fronto-striatal systems can compensate for some level of vulnerability or compromise to the system, whether from preexisting susceptibility or preliminary drug exposure. Evidence from other disorders also suggests compensatory recruitment in key dmPFC regions associated with response-inhibition (de Wit et al., 2012). However, with increasing usage or greater vulnerability compensation is no longer possible, resulting in reduced neural recruitment and disrupted performance. Numerous studies have also indicated excessive cerebellar recruitment during response-inhibition in impulsive groups including SDI (Connolly et al., 2012, Hester and Garavan, 2004, Rubia et al., 2013, White et al., 2014). Both unaffected siblings and recreational users share increased cerebellar grey matter density (Ersche et al., 2013b). Thus, complementary compensatory routes may exist when fronto-striatal mediation of response-inhibition becomes sufficiently compromised.

## 7 A broader view of disinhibition and stimulant dependence

Despite a superficially coherent picture, several issues merit discussion within the broader context of cognition and addiction models. First, chronic stimulant use has been associated with disruptions, not only in response-inhibition, but across a broad set of cognitive domains associated with fronto-striatal systems such as planning, working memory, decision-making and attention (Fernandez-Serrano et al., 2011, Jovanovski et al., 2005). Effect sizes are typically moderate and in this regard response-inhibition may serve as a representative assay of fronto-striatal integrity mediating top-down executive control (Friedman et al., 2008). Nevertheless, it is evident that impulsivity, compulsivity and executive function are fractionated and complex (Dalley et al., 2011, Miyake et al., 2000). Thus it may be of use in future to extend their ecological validity by examining how combining multiple constructs, such as response-inhibition and impulsive choice, may contribute to drug dependence symptomatology, as evidenced in other clinical populations (Solanto et al., 2001).

Another issue is polysubstance use, which is an inherent part of the common SDI phenotype (Pennings et al., 2002). Response-inhibition performance is likely disrupted in alcohol dependence and possibly nicotine dependence per se, at least under conditions of withdrawal, though it is unclear again whether disruptions are specific to response-inhibition (Fillmore and Vogel-Sprott, 1999, Goudriaan et al., 2006, Kozink et al., 2010, Monterosso et al., 2005, Mulvihill et al., 1997, Nederkoorn et al., 2009). This raises the possibility that it is disrupted across various substance dependence categories, along with mediating fronto-striatal circuitry interferences. Alternatively, it has been proposed that stimulants and alcohol have a more specific effect on impulsive action, including response-inhibition, compared to other drugs (Fernandez-Serrano et al., 2011). Research on pure recreational stimulant users or on suitable animal models may help disentangle the effects of stimulants in relation to alcohol and nicotine.

Response-inhibition difficulties are also apparent across a broad spectrum of neuropsychiatric conditions (Robbins et al., 2012). Moreover, SDIs are characterized by a high rate of comorbid personality and mood disorders (Cheetham et al., 2010). ADHD is particularly of interest to addiction research given shared ateological influences, common dopaminergic involvement, and overlapping fronto-striatal abnormalities, such as vlPFC hypoactivation associated with impaired response-inhibition (Hart et al., 2013). Children with ADHD are also significantly more likely to develop substance use disorders (Lee et al., 2011). Increased impulsivity, manifested in part by impaired response-inhibition and common to both groups, drives the similarities as they are evident in each patient group without comorbidities (Morein-Zamir et al., 2013, Morein-Zamir et al., 2014). Disruptions to the fronto-striatal circuits can also be found in disorders of excessive compulsivity such as OCD and trichotillomania and first-degree relatives of the former (Chamberlain et al., 2007b, Chamberlain et al., 2007c). In fact, we anticipate that any disorder characterized by executive function weakness and disrupted fronto-striatal circuity should demonstrate performance disruptions in response-inhibition, as in Parkinson’s disease and Tourette’s disorder (Gauggel et al., 2004, Goudriaan et al., 2006, van den Wildenberg et al., 2006). Some specificity is apparent as such difficulties have not been found in anxiety disorders (Lipszyc and Schachar, 2010).

Research to date has not revealed a strong link between response-inhibition as measured in GNG and SST per se and OFC and vmPFC integrity, despite both regions being implicated in compulsivity (Milad and Rauch, 2012) and addiction (Koob and Volkow, 2010). Turning to OCD as a key disorder of compulsivity, both OCD patients and SDI are characterized by increased ritualistic, time consuming compulsive behaviors, where OFC abnormalities are considered key (Meunier et al., 2012, Rotge et al., 2008). OFC and dorsomedial striatum integrity is implicated in mediating cognitive flexibility and suppressing a previously rewarded response in favor of an alternative (Chamberlain et al., 2008, Jentsch et al., 2014). Reduced OFC grey matter density and metabolism is found in SDI and, in early adolescents, is predictive of subsequent illicit substance use (Cheetham et al., 2010, Whelan et al., 2012, though see Montigny et al., 2013). OFC and vmPFC circuits could mediate complementary mechanisms underlying dependence symptomatology, such as rigidity, perseverative responding and increased habit formation (Jentsch et al., 2014, Voon et al., 2014). Whether response-inhibition contributes in some indirect manner to dysfunction in these seemingly independent mechanisms, or interacts with them is an open question, although the interconnectedness of fronto-striatal circuits suggests this possibility (Milad and Rauch, 2012).

## 8 Future directions

Response-inhibition is one of several complex interacting processes such as impulsive choice, altered decision making, salience attribution and motivational/reward and emotional dysregulation which contribute to the dependence phenotype (Cheetham et al., 2010, Goldstein and Volkow, 2011, Koob and Volkow, 2010). Both human and rodent models of response-inhibition and top-down control relevant to substance dependence are undergoing refinement, as integration from multiple lines of independent enquiry better approximate phenotypic complexity. Inhibitory dyscontrol contributes to substance dependence via increased impulsivity and compulsivity and general executive function weakness constituting overlapping routes, all undergoing changes due to drug exposure and natural maturation. Given the progress in understanding response-inhibition, it can serve as a particularly useful and valid model of how maturation as well as gender and genetic interactions influence fronto-striatal mediated top-down control (Li et al., 2006a, Nymberg et al., 2013, Rahman and Clarke, 2005, Rubia et al., 2013, White et al., 2014). Disruptions of behavioral inhibition and executive control are mechanisms through which lower socioeconomic status (Hackman and Farah, 2009) and early stress may lead to reported social difficulties (Volkow et al., 2011) and increased likelihood of exposure to drug and transitions to dependence. Thus, there is a need for integration of research between top-down executive control, altered reward systems, and motivational mechanisms, possibly relating to factors such as negative urgency, affect and stress to inhibitory control in substance dependence (Albein-Urios et al., 2013, Cheetham et al., 2010, Li and Sinha, 2008). For example, how does impulsive choice coupled with reduced response-inhibition lead to symptoms? While each line of enquiry is supported by a rich body of findings, examining how response-inhibition during temporal unfolding of substance dependence, is influenced by acute stress, changes in motivation and affect can advance this goal and promote its clinical relevance. The modulation of response-inhibition by affect and motivation (Morie et al., 2014a, Pike et al., 2013) may provide converging evidence to lines of enquiry into cognitive control focusing on drug-related materials such as the emotional Stroop (Smith et al., 2013).

How might better understanding of response-inhibition be of relevance to treatment of SDI? From a conceptual standpoint, cognitive difficulties, although largely of medium effect size, may have pronounced consequences on daily functioning and can negatively affect treatment outcome and prevention. General inhibitory control impairments tend to be associated with poor treatment retention and increased dropout (Aharonovich et al., 2008, Stevens et al., 2014). From a conceptual standpoint, proposed therapies strengthening inhibitory function should benefit from the strong preclinical and cognitive neuroscience evidence. Accordingly, cognitive behavioral therapy is believed to lead to improvements at least in part by influencing executive functioning (Sofuoglu et al., 2013). Treatment approaches could be allocated based on individual cognitive function also accounting for the impulsive cognitive style characteristic of SDI (Stevens et al., 2014). Applying pharmacological agents with low abuse potential to improve cognitive functioning and specifically response-inhibition could thus be doubly useful. Recent studies have proposed that modafinil may facilitate clinical improvement during initial abstinence via blunting cocaine-induced euphoria (Dackis et al., 2005) or normalizing disrupted sleep patterns (Morgan et al., 2010). Improved top-down control may be another mechanism by which such treatments may operate (Economidou et al., 2009). In healthy individuals and in ADHD patients modafinil and atomoxetine have improved aspects of cognition, including response-inhibition (Chamberlain et al., 2006, Chamberlain et al., 2007a, Turner et al., 2003, Turner et al., 2004). In fact, atomoxetine, licensed for ADHD treatment, was found to exert its beneficial effects on response-inhibition via modulation of the right vlPFC (Chamberlain et al., 2009). However, despite initial promise for modafinil, it appears that comorbid alcohol use as well as gender and concomitant behavioral therapy may be important qualifiers for effectiveness in relapse prevention (Anderson et al., 2009, Dackis et al., 2012). Similarly, a recent study of atomoxetine in SDI found no significant improvement, although it is possible these patients had already well-established habits (Walsh et al., 2013).

Present evidence regarding neurostimulation, either via repetitive transcranial magnetic stimulation or transcranial direct current stimulation, has focused on the DLPFC circuit as a viable therapeutic procedure for improving symptoms such as craving (Jansen et al., 2013). Evidence that pre-SMA or vlPFC neurostimulation may improve response-inhibition is still preliminary (Ditye et al., 2012, Jacobson et al., 2011), but could serve as an additional therapeutic target. As the role of individual differences is elucidated, impaired response-inhibition may provide a well understood mechanism allowing for the prediction of the best responders to such interventions, possibly in combination with improved behavioral therapies.

The field has benefitted thus far from translational cross-species paradigms, with response-inhibition constituting a key element of the impulsivity construct. Use of such paradigms in experimental animals is highlighting a fractionation of the impulsivity construct in terms of fronto-striatal circuits (Dalley et al., 2011) implying a different view of how response inhibitory executive control is implemented. An important step will now be to test this approach in humans, including SDI. For example, Voon et al. (2014) recently showed in an analogue of the rodent 5-choice task that methamphetamine abusers and recreational cannabis users both exhibited higher levels of impulsive responding. We predict that a combination of investigations in both experimental animals and in humans will be required to fully understand the role of response inhibitory impairments in stimulant dependence and addiction more generally.
